# Effects of different granulocyte colony stimulating factor regimens on patients with euploid embryo transfer in recurrent implantation failure

**DOI:** 10.3389/fmed.2025.1583385

**Published:** 2025-05-26

**Authors:** Ozge Karaosmanoglu, Aysen Yuceturk, Ilke Ozer Aslan, Sule Yildirim Kopuk, Zeynep Ece Utkan Korun, Yigit Cakiroglu, Bulent Tiras

**Affiliations:** ^1^Assisted Reproductive Technologies Unit, Acibadem Maslak Hospital, Istanbul, Türkiye; ^2^Assisted Reproductive Technologies Unit, VM Medical Park Pendik Hospital, Istanbul, Türkiye; ^3^Department of Obstetrics and Gynecology, Yeditepe University, Istanbul, Türkiye; ^4^Department of Obstetrics and Gynecology, Acibadem Mehmet Ali Aydinlar University, Istanbul, Türkiye

**Keywords:** euploid embryo, granulocyte colony stimulating factor, *in vitro* fertilization, recurrent implantation failure, preimplantation genetic testing

## Abstract

**Objective:**

This retrospective cohort study aimed to investigate the effects of different colony-stimulating factor regimens in patients with recurrent implantation failure who underwent euploid embryo transfer.

**Methods:**

In total, 293 women with a history of recurrent implantation failure were included. The participants were divided into three groups: Group 1 received intrauterine granulocyte colony-stimulating factor (G-CSF) 5 days before embryo transfer; Group 2 received both intrauterine G-CSF and subcutaneous G-CSF from 5 days before embryo transfer until pregnancy; and Group 3 served as the control. Hormonal treatment included a 14-day regimen of oral estradiol followed by vaginal and intramuscular progesterone.

**Results:**

Primary outcomes included pregnancy, biochemical pregnancy, miscarriage, and live birth rates. The pregnancy rate (positive serum hCG) was significantly higher in group 2 compared to the control (65.9% vs. 50.5%). Group 1 had a higher pregnancy rate than the control, but the difference was not statistically significant (56.8% vs. 50.5%). Live birth rates were statistically significantly higher in Group 2 than Group 3 (55.7% vs. 40.6%). Group 2 had a higher live birth rate than group 1, but the difference was not statistically significant (55.7% vs. 47.3%). Although Group 1 had a higher live birth rate than Group 3, the difference was not statistically significant (47.3% vs. 40.6%).

**Conclusion:**

Addition of subcutaneous G-CSF to intrauterine injections may be associated with improved positive pregnancy test results and live birth rates in recurrent implantation failure.

## 1 Introduction

The diagnostic work-up during infertility has been rapidly progressing. Parallel with diagnostic methods, interventional modalities have also been widely used in assisted reproductive technologies (ART) (1). A “good quality” embryo in a “healthy endometrial microenvironment” does not always end up in a live birth. Additionally, it is difficult to determine the underlying factors that may interfere with implantation failure (2).

Recurrent implantation failure (RIF) is a debatable topic, with no consensus on its definition (3). Our suggestion for the definition of RIF includes the absence of implantation after cumulatively transferring four good-quality cleavage stages or blastocyst embryos (at most two embryos per transfer) within two fresh or frozen cycles diagnosed by a negative serum human chorionic gonadotropin test 14 days after embryo transfer (4). Possible etiological factors related to RIF might be the uterine microenvironment, embryonic factors, and factors acting on the interaction between the uterus and embryo. However, in most cases, the aetiology is not known to be unexplained recurrent implantation failure.

Preimplantation genetic testing for aneuploidy (PGT-A) was introduced to detect genetically abnormal embryos and rule out the transfer of a low implantation potential embryo (5). PGT-A is usually offered to couples with a history of chromosomally abnormal pregnancy, recurrent implantation failure (RIF), recurrent miscarriage, severe male factor infertility, and advanced maternal age (6). To increase live birth rates and transfer good-quality embryos, PGT-A has been suggested as an adjuvant modality in patients with RIF.

To improve IVF outcomes, Adjuvant immunotherapies have been suggested to improve IVF outcomes (7). Intrauterine infusion therapies have been investigated in the literature (8). Among the new immunological therapies on the impact of pregnancy outcomes, platelet rich plasma (PRP), peripheral blood mononuclear cells (PBMNC), G-CSF, and human chorionic gonadotropin have been shown to be the four most commonly used interventions (9). Granulocyte colony-stimulating factor has been shown to play a beneficial role in clinical outcomes after ET, especially in RIF cases (10). Granulocyte colony-stimulating factor (GCSF) is a member of the cytokine receptor superfamily and is synthesized in decidual cells (11–13). Granulocyte-Colony Stimulating Factor (G-CSF) is expressed in the fetomaternal area, placenta, decidua, and cervicovaginal fluids during the period appropriate for the implantation window and is an important marker in determining endometrial receptivity. G-CSF is a glycoprotein secreted by the endothelial cells, macrophages, and other immune cells. G-CSF plays a role in embryo implantation and continuation of pregnancy by temporarily suppressing the immune response and its effects on lymphocytes, macrophages, and T helper-2 cells (14). Its use may be associated with cytokine secretion, activation of T-regulatory cells, promotion of local immune responses, remodeling of the vascular endometrium, and recruitment of Th-2-promoting dendritic cells. When administered systemically, G-CSF has been reported to play a role in embryonic development, implantation, and trophoblastic growth, whereas local intrauterine administration can improve endometrial receptivity (15).

Studies on the effectiveness of subcutaneous or intrauterine G-CSF administration for RIF are limited. According to a meta-analysis published by Busnelli et al. (16) on the effect of intrauterine and subcutaneous G-CSF infusion in patients with RIF, subcutaneous administration of G-CSF was associated with an increased chance of clinical pregnancy compared to no G-CSF therapy (RR 2.29; 95% CI 1.58 to 3.31, 4) (RCT, *n* = 333). Intrauterine G-CSF administration had no effect on live birth rates (RR 1.53, 95% CI 1.00 to 2.33, 2 RCT, *n* = 257). A meta-analysis published by Hou et al. (17) reported that subcutaneous or intrauterine G-CSF administration increases clinical pregnancy rates. The initial method of G-CSF administration in routine practice is intrauterine infusion. After Hou et al.’s meta-analysis, we shifted our procedure to intrauterine and subcutaneous infusion simultaneously.

Our aim was to investigate the effects of different colony-stimulating factor regimens on IVF outcomes in euploid embryo patients with recurrent implantation failure. We also attempted to determine the most effective route of administration for patients with RIF.

## 2 Materials and methods

### 2.1 Study design and patient selection

This retrospective cohort study was conducted at the Acibadem University IVF Center in Istanbul, Turkey, between July 1, 2019, and July 1, 2023 (Clinical trial number: not applicable). Inclusion criteria: age 20–40, transfer of one PGT-A euploid blastocyst, ≥2 prior failed embryo transfers with good-quality embryos. Patients with untreated uterine cavity abnormalities or other confounding treatments were excluded. A total of 293 patients met criteria. They were managed under evolving clinical protocols regarding G-CSF use (no randomization). Group 1 (Intrauterine G-CSF): intrauterine infusion of 300 μg filgrastim 5 days before embryo transfer. Group 2 (Combined Intrauterine + Subcutaneous G-CSF): intrauterine G-CSF as in Group 1 plus subcutaneous G-CSF (300 μg) 5 days before transfer and on the day of transfer, with weekly injections continued until a pregnancy test and early pregnancy if positive. Group 3 (Control): no G-CSF. Oral consent was obtained from all patients who were informed of the use of their medical records, including medical history, family history, fertility treatments, and outcomes, as well as their pregnancy and delivery outcomes and follow-ups. Those who did not consent to participate or were unable to reach a consensus were excluded from the study. Other exclusion criteria were as follows: chromosomal and genetic abnormalities; congenital or acquired (fibroid, polyp, synechia) uterine abnormalities; endometriosis, adenomyosis, chronic diseases (HT, DM, SLE, thyroid diseases), and thrombophilia. The study protocol was approved by the Institutional Review Board and Ethics Committee of Acibadem Mehmet Ali Aydınlar University (ATADEK-2022-20/04) and the research was conducted in accordance with the Declaration of Helsinki. The pregnancy outcomes of patients were monitored until July 2023.

We retrospectively analysed the data of patients with a 1:1 ratio in each group. Group 1: Intrauterine G-CSF (Neupogen, Roche, Istanbul, Turkey, 48 MIU/0.5 mL) was administered 5 days before embryo transfer; Group 2: Patients who were administered intrauterine G-CSF in addition to subcutaneous (0.1 mg/kg) G-CSF five days before embryo transfer and applied until pregnancy test; Group 3: Patients who were not administered G-CSF will be included in the control group. Controlled ovarian hyperstimulation (COH) was initiated on the second or third day of the spontaneous or induced menstrual cycles. Gonadotropin stimulation was initiated at a dose of 300 IU of recombinant FSH (Gonal F; Merck, or Fostimon; IBSA) and 300 IU human menopausal gonadotropin (hMG) (Merional; IBSA). As soon as the dominant follicle reached a mean diameter of 14 mm, a GnRH antagonist (Cetrotide; Merck) was initiated. When at least one leading follicle reached a mean diameter of 18 mm, 250 μg of recombinant choriogonadotropin alfa (rHCG, Ovitrelle; Serono) was administered to induce final follicle maturation.

Oocyte retrieval was performed 36 h after rhCG administration. Four hours after retrieval, oocyte denudation was performed, and all mature oocytes were inseminated via ICSI. Based on patient and physician preferences, good-quality embryos were either transferred on day 3 or day 5 after oocyte retrieval. For patients who opted for preimplantation genetic testing for aneuploidy (PGT-A), all good-quality blastocysts underwent trophectoderm biopsy followed by vitrification. PGT-A was performed in a commercial laboratory by using an NGS-based assay (Veriseq PGS). Samples were reported as “euploid,” “aneuploid,” “mosaic,” or “no result.” Only euploid embryos were transferred after PGT-A treatment. A single FET was scheduled 116–120 h after progesterone initiation.

The primary outcomes included a positive pregnancy test rate and live birth rate. Secondary outcomes assessed were biochemical pregnancy rate and miscarriage rate.

### 2.2 Pregnancy outcomes

Pregnancy outcome was determined 12 days after ET by assessing serum ß-hCG levels. Clinical pregnancy was defined as the presence of a gestational sac or fetal pole on transvaginal ultrasonography after a positive pregnancy test. Live birth rate was defined as the number of deliveries, resulting in a live born neonate after 24 weeks of gestation. Miscarriage was defined as the loss of pregnancy before 12th weeks of gestation.

### 2.3 Statistical analysis

All data were analysed using SPSS (SPSS-IBM 2.3, Inc., Chicago, IL, USA) and MedCalcsoftware version 18.11.6 (MedCalc Software, Broekstraat 52, 9,030 Mariakerke, Belgium). The Shapiro–Wilk test was used to assess data normality. For the matched samples of GCSF before and after measurements, in addition to descriptive statistics, a one-way ANOVA test, Fisher’s exact test, and categorical variables (% per group) were compared with Chi-square or Fisher’s exact test (chi-square or Fisher’s exact for categorical outcomes such as pregnancy rates, and one-way ANOVA for continuous variables like age and BMI, with post-hoc tests as appropriate). For continuous variables, the study results were summarized as mean± standard deviation (SD). Categorical variables were presented as frequencies and percentages. Categorical variables were presented as frequencies and percentages. Statistical significance was set at *p* < 0.05; categorical variables (% per group) were compared with Chi-square or Fisher’s exact test. Significance was set at *p* < 0.05. No pre-study sample size calculation was performed (retrospective design), but we conducted a post-hoc power analysis. Based on observed group differences in pregnancy rates, the sample size provided ~85% power (*α* = 0.05) to detect the difference between combined G-CSF and control.

## 3 Results

A total of 293 women with a history of recurrent implantation failure after hormone replacement therapy for FET of a euploid embryo were included in the study. There were 95 patients in the intrauterine G-CSF group (group 1), 97 in the intrauterine + subcutaneous G-CSF group (group 2), and 101 in the control group (group 3). Table 1 shows baseline characteristics. The three groups were similar in age (Group 1: 33.2 ± 3.0; Group 2: 33.6 ± 3.7; Group 3: 33.1 ± 3.2 years), partner’s age, BMI, infertility duration, and number of prior failed cycles (*p* > 0.05 for all). There were no significant baseline differences, indicating the groups were comparable. The age range was 30–40 (33.3 ± 3.3). None of the groups differed in mean age, partner’s age, BMI, infertility duration, number of previous IVF trials, total antral follicle counts, number of days of stimulation, total gonadotropin dose, number of retrieved oocytes, number of blastocysts, or endometrial thickness on the start day (Table 1).

**Table 1:** Comparison of sociodemographic parameters and variables in between the groups.

	Group 1 (*n* = 95)	Group 2 (*n* = 97)	Group 3 (*n* = 101)	*P*
Age (years)	33.2 ± 3.0	33.6 ± 3.7	33.1 ± 3.2	*
Partner’s age (years)	35.9 ± 5.4	36.1 ± 5.6	35.7 ± 5.7	*
BMI (kg/m^2^)	25.6 ± 5.1	26.2 ± 4.7	25.1 ± 3.7	*
Infertility duration (months)	80.8 ± 47.2	77.7 ± 48.4	86.9 ± 55.2	*
Number of previous IVF trials	2.7 ± 0.9	2.5 ± 0.0.9	2.6 ± 1.2	*
Total antral follicle counts (n)	18.8 ± 10.4	18.3 ± 10.5	18.6 ± 12.8	*
Days of stimulation (n)	10.4 ± 1.5	10.2 ± 1.4	10.0 ± 1.3	*
Total gonadotropin dose (IU)	3,112 ± 439	3,056 ± 429	3,000 ± 404	*
Number of oocytes retrieved (n)	11.8 ± 5.1	11.6 ± 5.5	11.9 ± 7.0	*
Number of blastocysts (n)	5.8 ± 1.8	5.8 ± 1.5	5.3 ± 2.2	*
Endometrial thickness on P start day	10.9 ± 2.6	11.1 ± 2.3	10.5 ± 1.9	*

* No statistical significance.

Pregnancy outcomes of the groups were summarized in Table 2. Pregnancy, defined as a positive serum hCG test, was significantly higher in the intrauterine and subcutaneous groups (group 2) compared to the control group (65.9% vs. 50.5%; *p* = 0.031). The intrauterine G-CSF group (Group 1) had higher positive hCG test than the control group (Group 3) and lower positive hCG test compared to Group 2, with no statistical significance (56.8% vs. 50.5%; *p* = 0.393 and 56.8% vs. 65.9%; *p* = 0.236, respectively). The biochemical pregnancy rates were similar in all groups, without statistically significant (5.3% vs. 5.2% vs. 4.0%, respectively). The Miscarriage rates were also similar in all groups, with no statistical significance (4.2% vs. 5.2% vs. 5.9%, respectively). Live birth rates were compared between groups. Group 2 had a statistically significantly higher rate compared than group 3 (55.7% vs. 40.6%; *p* = 0.046). The live birth rate was also higher in group 2 compared to group 1, but the difference was not statistically significant (55.7% vs. 47.3%; *p* = 0.046). Although the live birth rate was higher in Group 1 compared to Group 3, the difference was not statistically significant (47.3% vs. 40.6%; *p* = 0.388). All pregnancies were singleton.

**Table 2:** Comparison of pregnancy outcomes between the groups.

	Group 1 Intrauterine (*n* = 95)	Group 2 Intrauterine+Subcutaneous (*n* = 97)	Group 3 Control (*n* = 101)	*P*
Positive hCG test	54/95 (56.8)	64/97 (65.9)	51/101 (50.5)	(0.031)*
Biochemical pregnancy	5/95 (5.3)	5/97 (5.2)	4/101 (4.0)	NS
Miscarriage	4/95 (4.2)	5/97 (5.2)	6/101 (5.9)	NS
Live birth	45/95 (47.3)	54/97 (55.7)	41/101 (40.6)	(0.046)*

* Statistically significant difference between Group 2 and Group 3.

NS, Not statistically significant.

## 4 Discussion

To our knowledge, this is the first study to investigate the effects of different routes of G-CSF administration on IVF outcomes in patients with RIF who underwent euploid embryo transfer. Zeyneloglu et al. (18) analysed the effects of G-CSF administration in patients with RIF. They compared the results in patients with subcutaneous administration, both subcutaneous and intrauterine administration (dual administration), with the control group. They have reported highest live birth rates in the dual administration group, followed by only the subcutaneous G-CSF group, with the lowest rates in the control group (61.5% vs. 34.2% vs. 23.5%; *p* = 0.001, respectively).

G-CSF administration has been investigated in patients with infertility and recurrent pregnancy losses (19). The thin endometrium and recurrent implantation failure have been the main focus of interest in infertile patients treated with G-CSF (20). Fu et al. (21) analysed 14 studies including 1,387 patients with RIF, revealing increased clinical pregnancy rates in both fresh and frozen ET cycles (RR 1.58; 95 CI, 1.18–2.11; and RR 1.98; 95 CI, 1.55–2.54, respectively). In a meta-analysis of six studies, higher clinical pregnancy, implantation, and biochemical pregnancy rates were reported compared to placebo (RR:1.563, 95% CI: 1.122, 2.176; RR:1.887, 95% CI: 1.256, 2.833; RR:2.385, 95% CI: 1.414, 4.023, respectively) (22). In another systematic review and meta-analysis, two trials on RIF were analysed (23). Administration of G-CSF has been reported to be associated with significantly higher clinical pregnancy rates compared with the no intervention group, with a low quality of evidence (RR:2.51; 95 CI:1.36–4.63; I2 0%).

Subcutaneous G-CSF administration has also been investigated. One of the earliest datasets about patients with RIF comes from Würfel et al. (24). They performed an RCT investigating the role of G-CSF in implantation with 300 μg of G-CSF on the embryo transfer day. Their results revealed higher pregnancy rates in the G-CSF group compared to the control group (50.7% vs. 19.8%). Scarpellini et al. (25) reported a randomized controlled trial about G-CSF treatment in 109 women with RIF. They administered 60 mg/day subcutaneous G-CSF from the day of transfer to the day of the hCG test (if the result was positive, G-CSF was continued for another 40 days) to the intervention group and treated the patients in the control group in the same way as the subcutaneous saline solution. They reported statistically significantly higher pregnancy rates in the G-CSF group than in the control group (43.1% vs. 21.6%, *p* < 0.001). Abedi et al. (26) evaluated the efficacy of subcutaneous G-CSF in 100 infertile women with repeated implantation failure. They reported statistically significantly higher implantation and chemical pregnancy rates in the 300 μg G-CSF group compared to the control group (15.3% vs. 7.2 and 40% vs. 20%, respectively). Aleyasin et al. analyzed the effects of G-CSF in 112 patients with RIF in an RCT (27). They reported significantly higher implantation rates and chemical and clinical pregnancies with 300 μg of subcutaneous G-CSF administration (OR:2.63; OR:2.74, and OR:2.94, respectively). Arefi et al. (28) investigated pregnancy outcomes in patients with a history of unexplained RIF with 300 μg subcutaneous G-CSF administration 30 min before blastocyst transfer. Their results revealed higher clinical pregnancy and live birth rates without statistical significance (56.2% vs. 40.0%; 53.1% vs. 35.0%, respectively).

Other interventions for IU have also been reported in the literature. Davari-Tanha et al. (29) investigated the role of G-CSF in patients with RIF in a double-blind, randomized controlled trial in both fresh and frozen cycle. They administered 300 μg of G-CSF on the day of oocyte retrieval or on the day of progesterone administration in the FET cycle. Their results indicated higher implantation rates in the G-CSF group compared to the saline and control groups (12.3, 6.1, and 4.7%). Eftekhar et al. (30) analyzed the effect of intrauterine infusion of 300 μg of G-CSF in patients. They reported higher clinical pregnancy rates compared to the control group (28.8% vs. 13.3%; *p* = 0.043). Huang et al. reported reduced miscarriage rates and improved live birth rates in a prospective randomized single-blind trial of FET cycles (31). Jalilvand et al. (32) demonstrated the efficacy of uterine G-CSF injections in patients. Contrary to other publications, they did not report any significant difference between the intervention and control groups with regard to gestational sac with observed fetal heart rate and positive serum hCG test results. Kalem et al. (33) published an RCT comparing the effect of G-CSF once daily on hCG with saline as a control group in fresh ET cycles. They reported similar clinical pregnancy rates, miscarriage rates, and live birth rates between the G-CSG and control groups, which differed from previous studies (*p* = 0.112, *p* = 0.171, *p* = 0.644, respectively).

The mechanisms underlying the benefit of combined G-CSF likely involve both local uterine effects and systemic immune modulation. Intrauterine G-CSF can directly improve the endometrium – prior studies show it can increase endometrial thickness and alter expression of implantation-related genes (21). Subcutaneous G-CSF enters systemic circulation, potentially influencing the immune cells (like uterine natural killer cells and T cells) to create a more hospitable environment for the embryo. By temporarily suppressing detrimental immune responses and promoting a tolerant Th2-biased milieu, G-CSF may prevent immune rejection of the embryo and support trophoblast invasion (34). Our combined regimen presumably provided both benefits: the intrauterine infusion primed the endometrium locally, and the ongoing subcutaneous dosing maintained systemic support during the implantation window and early pregnancy. The significantly higher success rates in the combined group support a hypothesis of a synergistic effect. While we cannot conclusively prove synergy without a subcutaneous-only group for direct comparison, the data trend suggests dual administration could be more effective than a single route.

We emphasize that a prospective randomized trial is needed to confirm the efficacy of combined G-CSF, eliminating selection bias. Such a trial could randomize RIF patients to combined G-CSF vs. single-route vs. placebo to clearly delineate the added value of dual-route. We also highlight exploring optimal timing and dosage of G-CSF. Our regimen started 5 days pre-transfer; perhaps starting closer to transfer (or even on the day of transfer) could be equally or more effective, as one meta-analysis suggests ET-day administration yields good results (35). The dosage of G-CSF should also be optimized; it’s possible that lower doses could be used effectively or that only a couple of doses around the implantation window are needed, which could reduce cost and side effects.

It is important to interpret these results with an understanding of the study’s limitations. Selection bias is inherent to the retrospective design. The groups were not randomized; thus, there may be biases in which patients received G-CSF. We mitigated this by showing baseline factors were similar and by including all consecutive cases, but unknown confounders could still be present. We have now explicitly acknowledged this limitation and the need for caution in drawing causal inference. Additionally, this was a single-center study; though protocols were consistent internally, practices elsewhere may differ, and patient populations could vary. Our use of only euploid embryos is a strength (controlling embryo quality) but also means these findings specifically apply to RIF in the context of chromosomally normal embryos.

Additionally, it is very important to discuss cost-effectiveness analysis in future studies. G-CSF is relatively costly, and adding potentially multiple injections can increase the cost of an IVF cycle significantly. It is important to determine if the improvement in outcome justifies this cost. If combined G-CSF can substantially raise live birth rates for RIF patients, it may be cost-effective by reducing the need for further IVF cycles. On the other hand, if the benefit is modest, the cost may not be justified outside of research settings. We now explicitly suggest that future trials incorporate economic evaluations.

In conclusion, our results revealed that subcutaneous G-CSF, in addition to intrauterine injection, might be associated with improved positive pregnancy test results and live birth rates in women with a history of recurrent implantation failure. However, this intervention should be studied in prospective randomized clinical trials before wider clinical applications.

## Data Availability

The datasets presented in this article are not readily available because the datasets used and/or analyzed during the current study are available from the corresponding author on reasonable request due to the patient privacy concern. Requests to access the datasets should be directed to Yigit Cakiroglu, dryigit1@yahoo.com.
